# The Benefits of Self-Transcendence: Examining the Role of Values on Mental Health Among Adolescents Across Regions in China

**DOI:** 10.3389/fpsyg.2021.630420

**Published:** 2021-02-17

**Authors:** Ping Liu, Xiaofeng Wang, Dan Li, Rongwei Zhang, Hui Li, Jingxin Han

**Affiliations:** ^1^Department of Psychology, Shanghai Normal University, Shanghai, China; ^2^Center for Psychological Consultation, Shanghai University of Political Science and Law, Shanghai, China; ^3^Fujian Provincial Party School of the Communist Party of China, Fuzhou, China

**Keywords:** existential positive psychology, self-transcendence, COVID-19, values, mental health, adolescents

## Abstract

As one of the foundations of existential positive psychology, self-transcendence can bring positive intrapersonal and interpersonal outcomes, especially in the COVID-19 era in which people are suffering huge mental stress. Based on Schwartz's theory of human basic values, the current study combines variable-centered and person-centered approaches to examine the relationships between adolescents' values and mental health across two regions in China. The results generally showed that (1) both self-enhancement and conservation values were positively correlated with depression and loneliness, while both self-transcendence and openness to change values negatively correlated with depression and loneliness. The results also showed that (2) there were four value clusters (i.e., *self-focus, other-focus, anxiety-free, undifferentiated*), and, compared to adolescents in the *self-focus* and *undifferentiated* values cluster, all adolescents in the *anxiety-free* values cluster reported lower depression and loneliness, while all adolescents in the *other-focus* values cluster reported higher depression and loneliness. The differences between the two regional groups only emerged in depression. Specifically, adolescents in Shanghai have higher levels of depression than adolescents in Qingdao. This study provides some evidence for the new science of self-transcendence among adolescents and also sheds light on how we may improve the level of mental health during the COVID-19 era.

## Introduction

When comes to negative emotions, such as depression and loneliness, mental health experts always consider medication and cognitive behavior therapy as treatment options. However, other approaches, such as Viktor Frankl's theory of self-transcendence, provide a promising framework to explore how to achieve well-being through self-transcendence. Based on Frankl's self-transcendence model, Wong (2016) has developed existential positive psychology, which suggests that self-transcendence provides a way to achieve virtue, happiness, and meaning. Self-transcendence included three levels: (1) seeking the ultimate ideals of goodness, truth, and beauty; (2) being mindful of the present moment with an attitude of openness and curiosity; (3) seeking one's calling, which involves engagement and striving to achieve a concrete meaning in life—a life goal of contributing something of value to others (Wong, 2013). All three levels of self-transcendence are motivated by the intrinsic need for spiritual values (Wong, 2013). Thus, if one can cultivate healthy values, they will develop a healthy lifestyle that is good for individuals and society.

Values refer to what people find important in life, which are abstract and desirable goals that form an organized system to identify groups and individuals (Schwartz, 1992). Adolescence is a key period for the formation and development of values (Inglehart, 1978), and the values cultivated in adolescence have a direct impact on adolescents' adjustments (Sortheix and Schwartz, 2017; Benish-Weisman et al., 2019). In particular, the COVID-19 pandemic poses a significant threat to human's physical health and mental health. For example, during the epidemic period, the level of anxiety and depression of Chinese people has increased (Dong et al., 2020; Zhen and Zhou, 2020). Adolescents' study and life were also seriously affected by the epidemic. Schools in China have been forced to close, and students were urged to stay at home. As a result, they lacked physical exercises, peer communication, and had to face academic pressure alone; such “isolation” may increase their loneliness and depression.

Overall, this study aims to explore the influence of values on adolescents' mental health to provide some empirical evidence for how self-transcendence contribute to meaningful living and well-being during the COVID-19 era.

### Theory of Values and Mental Health

In the field of psychology, Schwartz's theory of human basic values is currently the most widely used model. Schwartz defines 10 values that are divided into four higher-order dimensions. The 10 values are organized around a circular continuum according to the motivation they expressed. Values that express similar motivations are located on the adjacent sides and can be pursued simultaneously. However, values that express conflicting motivations are located on the opposite sides of the circle and cannot be pursued simultaneously (Cieciuch et al., 2015).

There are two organizing principles to contrast these values (Schwartz, 2015). The first principle is the interests that value attainment serves. Personal-focus values emphasize the pursuit of one's own interests and relative success and dominance over others. In contrast, social-focus values emphasize concern for the welfare and interests of others. The second principle is the relationship between values and anxiety. Anxiety-free (i.e., self-growth) values express growth and self-expansion, which may lead to feelings of meaning and satisfaction. In contrast, anxiety-avoidance (i.e., self-protection) values express the need to avoid or control anxiety and threat and to protect the self, which may lead to negative emotions, such as depression and anxiety.

Openness to change values are personal-focus and anxiety-free, and express growth motivation and independence of thought, action, and feelings, and readiness for change. In contrast, conservation values are social focus and anxiety avoidance, which emphasize order, self-restriction, preservation of the past, and resistance to change. Although conservation values may promote social relations (Schwartz, 2015), adolescents are exploring and creating their own identity, and openness to change can satisfy their autonomy and independent needs. If such needs are met, adolescents will feel less depression and loneliness (Emery et al., 2015; Majorano et al., 2015).

Self-enhancement values are personal-focused and anxiety-avoidance, which emphasize the pursuit of self-interest by controlling others and resources or gaining social recognition and may increase aggression, which damages harmony relationships (Benish-Weisman, 2019). In contrast, self-transcendence values are social-focus and anxiety-free, which express a growth motivation and reflect care for others' well-being over self-interests and thus may increase prosociality, which helps to build and maintain relations. Harmony relationships help to increase positive emotions (Vaillant, 2002; Davis et al., 2018).

Wong (2013) pointed out that Schwartz's model of universal values may be useful for the self-transcendence construct. According to the type of goal or motivation that the value expresses, the openness to change and self-transcendence values are motivated by intrinsic needs (Schwartz, 2015). Behavior based on these values is rewarding in itself, providing satisfaction or pleasure through expressing autonomy and competence (openness to change values) or nurturance and relatedness (self-transcendence values) (Schwartz, 2015). Individuals who endorse the two values may be curious and open to novelty and may also be concerning the world and other people rather than on their own needs. And the self-transcendence model according to PP2.0 includes two main factors (1) benevolence to all people and (b) going beyond oneself to serve or connect with others or humanity, which are corresponding to the two subscales of Schwartz's value of self-transcendence. Thus, the self-transcendence values and openness to change values are in accordance with the meaning of self-transcendence, which is based on intrinsic motivation.

In addition, the present study examines values during adolescence because adolescence is a key period for the formation and development of values (Inglehart, 1978), and the values cultivated in adolescence have a direct impact on adolescents' behavior, attitude, and well-being (Ungvary et al., 2017; Bojanowska and Piotrowski, 2018; Seddig and Davidov, 2018). As indicators of psychological adjustment, depression and loneliness are common emotional problems in adolescence (Al-Yagon, 2011; Chen et al., 2012), but the question of whether values relate to mental health in adolescence has received little attention. Although studies on adults found some meaningful results (e.g., people who endorsed power values feel more worried) (Heim et al., 2019), value endorsement has significant differences between different age groups (Gouveia et al., 2015). Thus, it is necessary to further explore values and mental health among adolescents. More importantly, this study may extend the application of the self-transcendence from counseling therapy to normal adolescents, thereby helping adolescents to increase meaningfulness and well-being.

### Values Clusters and Mental Health

Previous studies have usually used traditional approaches (i.e., variable-centered approaches) to examine bivariate associations between values and behavior or psychological adjustments (Sortheix and Schwartz, 2017; Benish-Weisman et al., 2019). These studies have shed light on the influence of values; however, Schwartz (2015) noted that values represent a circular continuum of related motivations rather than a set of discrete motivations. To the best of our knowledge, two studies have used the new approach (i.e., a person-oriented approach) to group together people who have similar sets of dominating values and compared them in terms of psychological well-being (Bojanowska and Piotrowski, 2018) and aggression (Ungvary et al., 2017). Specifically, Ungvary et al. (2017) not only found that self-enhancement values positively correlated with aggression but also found that adolescents who high on self-enhancement and conservation values may have less aggression. Thus, such a person-centered approach helps to investigate the potential protective or destructive effect of one value on another (Benish-Weisman, 2019).

According to Schwartz's circle model, adjacent values, but not opposing values, will occur simultaneously. Four value clusters are identified: (1) *self-focused* values cluster high on openness to change and self-enhancement values; (2) *other-focused* values cluster high on self-transcendence and conservation values; (3) *anxiety-free* values cluster high on self-transcendence and openness to change values; (4) *anxiety-avoidance* values cluster high on self-enhancement and conservation values. The *anxiety-free* values cluster not only satisfies one's individual needs (i.e., autonomy and independence) but also one's interpersonal needs (i.e., positive relations). Wayment and Bauer (2017) suggested that quiet ego, which reflects a balance of concerns for the self and others, can promote one's well-being. Thus, the *anxiety-free* values cluster may be most adaptive during adolescence (Bojanowska and Piotrowski, 2018).

### Context Differences

Although the meaning of each value is universal across cultures and contexts (Schwartz and Bardi, 2001), the relationships between adolescents' values and adjustments may be moderated by contextual factors (Heim et al., 2019). For example, adolescents, in urban areas but not in rural areas, who value uniqueness have better peer relationships and academic performance (Chen et al., 2012). Sortheix and Lonnqvist (2014) found that self-orientation values were positively related to life satisfaction in low Human Development Index countries but negatively related to life satisfaction in high Human Development Index countries. Sortheix and Schwartz (2017) further found that self-oriented values were more positively related to subjective well-being but other-oriented values were more negatively related to subjective well-being in lowly than in highly egalitarian cultures. Hence, regional or contextual factors should be considered moderators when examining the relationships between value clusters and mental health.

The samples in the present study were from Shanghai and Qingdao, Shandong Province, China. Both Shanghai and Qingdao are located on the eastern coastal areas of China; however, there are some differences between them in terms of economy and culture. On the one hand, the per capita disposable income of Shanghai residents in 2019 was 69,244 yuan, while the per capita disposable income of Qingdao urban residents in 2019 was 50,817 yuan. On the other hand, with the rapid development of the economy, individualism is becoming increasingly popular, but traditional collectivism is declining in Shanghai. However, Qingdao is more influenced by Confucian culture, which originated in Shandong Province. Confucians emphasize abiding by social hierarchy order and caring for others (Chen et al., 2012). Such different economic and cultural environments may lead to different effects on adolescents' values and mental health. Individuals will feel higher well-being when their personal values are congruent with the prevailing value environment (Sagiv and Schwartz, 2000). Thus, *other-focused* value clusters may be more adaptive in Qingdao, but *self-focused* value clusters may be more adaptive in Shanghai.

### The Current Study

According to Wong's self-transcendence, this study aims to examine the relationship between values and mental health to provide some reference for coping with psychological health problems during the COVID-19 pandemic. Specifically, the variable-centered approach was adopted to preliminarily examine the bivariate relationships between four high-order values and mental health. We hypothesize that (1) both self-transcendence and openness to change values negatively correlate with depression and loneliness, and both self-enhancement and conservation values positively correlate with depression and loneliness. The person-centered approach was adopted to group adolescents who have similar sets of dominating values and compared them in terms of mental health across two regions in China. We also hypothesize that (2) adolescents in Shanghai who endorse *anxiety-free* value clusters or *self-focused* value clusters may feel less depression and loneliness, but adolescents in Qingdao who endorse *anxiety-free* or *other-focused* value clusters may feel less depression and loneliness.

## Methods

### Participants

Participants in the study consisted of 750 adolescents (381 males) were surveyed in November 2019 in Shanghai, and 823 adolescents (374 males) were surveyed in December 2019 in Qingdao, Shandong Province, China. The adolescents were in first and second grades in senior schools, with mean ages of 17.15 years (*SD* = 0.75) and 17.35 years (*SD* = 0.80) in the Shanghai and Qingdao groups, respectively. In addition, family background information is shown in Table 1, including parents' education level, parents' workplace, and whether they were only children. All participants were informed that the study was anonymous and voluntary, and they were given a gift as a reward for participation.

**Table 1:** Family background information.

		**Parents' education level**		**Parents' workplace**		**Only child or not**	
		**Junior school** **and below**	**Senior school** **and above**	**Local**	**Not local**	**Only child**	**Not only child**
Shanghai	Father	7.5%	92.5%	92.5%	7.5%	79.5%	20.5%
	Mother	10.6%	89.4%	97.9%	2.1%		
Qingdao	Father	51.3%	48.7%	95.6%	4.4%	32.4%	67.6%
	Mother	57.8%	42.2%	98.1%	1.9%		

### Measures

#### Values

The Portrait Values Questionnaire (PVQ; Cieciuch and Schwartz, 2012) was used to assess values. This questionnaire contains 40 items that describe a person's desirable goals that point to the importance of a type of value in an implicit way. For example, the item “*It is important to him/her to respond to others' needs. He/she tries to support those he/she knows*” describes a person who endorses benevolence values. Adolescents were requested to respond to 40 self-statements using a 6-point scale ranging from 1 (*not like me at all*) to 6 (*very much like me*). The PVQ has been used with adolescents in China [e.g., Chen et al. (2010) and Gu and Tse (2018)]. The internal reliabilities were 0.74, 0.78, 0.78, and 0.73 for the *self-enhancement, self-transcendence, openness-to-change* and *conservation* dimensions, respectively.

#### Depression

The 14-item Chinese version of the Children's Depression Inventory (Kovacs, 1992) was used to assess depression. Participants were asked to choose one response that best described him or her in the past 2 weeks from three alternative responses (e.g., “*I feel like crying every day*,” “*I feel like crying most days*,” and “*I feel like crying once in a while*”). Higher scores indicate greater depression. The measure has proven to be reliable and valid in Chinese adolescents [e.g., Li et al. (2018)]. The internal reliability was 0.85.

#### Loneliness

A self-report measure adapted from Asher et al. (1984) was used to assess loneliness. Adolescents were requested to respond to 16 self-statements (e.g., “*I have nobody to talk to*”) using a 5-point scale, ranging from 1 (*not at all true*) to 5 (*always true*). Higher scores indicate greater feelings of loneliness. The measure has been proven to be reliable and valid in previous studies conducted in Chinese children [e.g., Chen et al. (2016) and Coplan et al. (2017)]. The internal reliability was 0.93.

## Analytical Strategy

All analyses were conducted using SPSS Statistics 23.0 software.

First, to control for response tendency, a previous adjustment method (Schwartz et al., 2001; Gu and Tse, 2018) was followed to correct for individual differences in the use of the response scale of the Portrait Value Questionnaire. The following results were calculated after this adjustment. Thus, a positive value reflects prioritizing the values more than the average value importance to the person, and a negative value reflects prioritizing the value less than the average value importance to the person (Bardi et al., 2014).

Second, bivariate correlations were used to test relationships between values and depression/loneliness.

Third, K-means clustering was used to identify value clusters according to a previous study (Wang et al., 2015; Zhu et al., 2015; Bojanowska and Piotrowski, 2018). It is a strategy that divides samples into subsamples that share a common distribution of data. According to Bojanowska and Piotrowski (2018), a four-cluster solution was used in the present study.

Last, a 2 (Region) × 4 (Cluster) multivariate analysis of variance (MANOVA) was conducted to compare these four value clusters between two regions in terms of depression and loneliness.

## Results

### Common Method Bias Test

Harman's single-factor test was used to test the common method bias in this study. The common method bias means that one obtains only one factor that accounts for most of the variability when conducting exploratory factor analysis (EFA) (Zhou and Long, 2004). Based on the rotated principal component, EFA was conducted for all the variables, and 14 factors whose eigenvalues were >1 were extracted. The first factor accounted for 17.08%, far <40% of the variance. It can be concluded that there is no serious common method bias in this study.

### Bivariate Correlations Between Values and Mental Health

Means and standard deviations for values and mental health are presented in Table 2, along with correlations among the variables.

**Table 2:** Means, standard deviations, and correlations among values and mental health.

	***M***	***SD***	**1**	**2**	**3**	**4**	**5**	**6**
Self-enhancement	−0.42	0.65	1					
Self- transcendence	0.16	0.46	−0.55^**^	1				
Openness to change	0.26	0.52	0.03	−0.35^**^	1			
Conservation	−0.16	0.40	−0.39^**^	−0.03	−0.67^**^	1		
Depression	1.49	0.33	0.07^**^	−0.05^*^	−0.07^**^	0.06^*^	1	
Loneliness	2.08	0.68	0.04	−0.10^**^	−0.16^**^	0.21^**^	0.62^**^	1

* *p < 0.05, two-tailed*;

** *p < 0.01, two-tailed*.

Self-enhancement and conservation values correlated positively with depression, and self-transcendence and openness to change values correlated negatively with depression. Similarly, conservation correlated positively with loneliness, and self-transcendence and openness to change correlated negatively with loneliness.

### Values Clusters

K-means clustering was used to group our sample into subsamples of people with similar values. The final four value clusters created by K-means clustering are presented in Table 3. According to the bipolar dimensions in value theory (Schwartz, 2015) and a previous study (Ungvary et al., 2017), the first cluster, labeled *other-focus*, has higher levels of self-transcendence and conservation values than the other values. The second cluster, labeled *undifferentiated*, has relatively consistent levels of endorsement across the four values. The third cluster, labeled *self-focus*, has higher levels of self-enhancement and openness to change values than the other values. The fourth cluster, labeled *anxiety-free*, has relatively higher levels of self-transcendence and openness to change values than the other values. According to Bojanowska and Piotrowski (2018), the four clusters in our study differentiate value hierarchies well because for each value, at least one pair of groups differed.

**Table 3:** Four value clusters created by K-means clustering.

	***Other-focus*** **(*N* = 266;** ***n*_**Shanghai**_ = 106)**	***Undifferentiated*** **(*N* = 574;** ***n*_**Shanghai**_ = 271)**	***Self-focus*** **(*N* = 348;** ***n*_**Shanghai**_ = 184)**	***Anxiety-free*** **(*N* = 385;** ***n*_**Shanghai**_ = 189)**
Self-enhancement	−1.04	−0.19	0.28	−0.98
Self-transcendence	0.58	0.09	−0.26	0.35
Openness to change	−0.29	0.001	0.69	0.64
Conservation	0.25	−0.04	−0.53	−0.29

To verify the accuracy of the classification results of K-means clustering, discriminant analysis was carried out on the above results (see Table 4). The results showed that 262 participants were accurately predicted to be in the *other-focus* cluster (the accuracy rate was 98.5%), 565 participants were accurately predicted to be in the *undifferentiated* cluster (the accuracy rate was 98.4%), 347 participants were accurately predicted to be in the *self-focus* cluster (the accuracy rate was 99.7%), and 368 participants were accurately predicted to be in the *anxiety-free* cluster (the accuracy rate was 95.6%). Overall, 1,542 participants were predicted correctly, and the accuracy rate was 98%. These results support the reliability of the classification results of K-means clustering.

**Table 4:** Discriminant analysis of the four values clusters created by K-means clustering.

	**Predicted group of discriminant analysis**					
		**1**	**2**	**3**	**4**	**Total**
Clusters of	1	262 (98.5%)	2 (0.8%)	0 (0%)	2 (0.8%)	266
K-means	2	6 (1%)	565 (98.4%)	1 (0.2%)	2 (0.3%)	574
clustering	3	0 (0%)	1 (0.3%)	347 (99.7%)	0 (0%)	348
	4	9 (2.3%)	4 (1%)	4 (1%)	368 (95.6%)	385

*1 = other-focus, 2 = undifferentiated, 3 = self-focus, 4 = anxiety-free*.

### Values Clusters and Mental Health: Region as a Moderator

Depression and loneliness were entered into a 2 × 4 multivariate analysis of variance (MANOVA) with the factors Region and Cluster. Descriptive statistics are shown in Table 5.

**Table 5:** The scores on depression and loneliness in four clusters between two regions (*M, SD*).

	**Shanghai (*****n*** **=** **750)**				**Qingdao (*****n*** **=** **823)**			
	**1**	**2**	**3**	**4**	**1**	**2**	**3**	**4**
Depression	1.55 (0.33)	1.56 (0.35)	1.54 (0.35)	1.48 (0.34)	1.51 (0.35)	1.43 (0.29)	1.49 (0.32)	1.41 (0.27)
Loneliness	2.13 (0.71)	2.14 (0.68)	2.00 (0.69)	1.93 (0.66)	2.27 (0.79)	2.14 (0.63)	2.10 (0.66)	1.94 (0.59)

*1 = other-focus, 2 = undifferentiated, 3 = self-focus, 4 = anxiety-free*.

Significant main effects of Region and Cluster were found, Wilk's λ = 0.96 and, 0.97 *F*_(2, 1564)_ = 24.61 and *F*_(6, 3128)_ = 6.51, *ps* < 0.001, η_*p*_^2^ = 0.03 and 0.01, respectively. There were no significant interactions between Region and Cluster, Wilk's λ = 0.99, *F*_(6, 3128)_ = 0.96, *p* = 0.45.

Follow-up univariate analyses revealed that adolescents in Shanghai had higher scores on depression than adolescents in Qingdao, *F*_(1, 1565)_ = 16.70, *p* < 0.001, η_*p*_^2^ = 0.01, and there was no difference between the two regions on loneliness, *F*_(1, 1565)_ = 3.52, *p* = 0.061. In addition, follow-up univariate analyses showed that the main effect of cluster on depression was significant, *F*_(3, 1565)_ = 3.23, *p* = 0.02, η_*p*_^2^ = 0.006. Multiple comparisons with Bonferroni adjustment found that the depression scores of the *other-focus* cluster were higher than those of the *anxiety-free* cluster, *p* = 0.04, and there was no significant difference in depression between the other clusters, *p* > 0.05. Follow-up univariate analyses also showed that the main effect of Cluster was significant on loneliness, *F*_(3, 1565)_ = 10.51, *p* < 0.001, η_*p*_^2^ = 0.02. Multiple comparisons with Bonferroni adjustment found that the loneliness score of the *other-focus* cluster was higher than that of the *self-focus* cluster (*p* = 0.04) and *anxiety-free* cluster (*p* < 0.001), the loneliness score of the *undifferentiated* cluster was higher than that of the *anxiety-free* cluster (*p* < 0.001), and there was no significant difference in loneliness between the other clusters, *p* > 0.05.

## Discussion

In the current study, the variable-centered approach was adopted to examine the relationships between four dimensions of values and mental health, and the person-centered approach was adopted to group adolescents who have similar sets of dominating values and compared them in terms of mental health across two regions in China.

In general, the study found that (1) both self-enhancement and conservation values correlate positively with mental disorder, and both self-transcendence and openness to change values correlate negatively with mental disorder; (2) four values clusters were identified, namely, *other-focus, undifferentiated, self-focus* and *anxiety-free*; (3) all adolescents in the *anxiety-free* values cluster reported lower scores on depression and loneliness than those in other values clusters, and all adolescents in the *other-focus* cluster reported higher scores on depression and loneliness than those in other values clusters; adolescents in Shanghai have higher levels of depression than adolescents in Qingdao.

### Links Between Values and Mental Health

According to the bipolar dimensions in value theory (Schwartz, 2015), values with conflicting motivations can lead to opposite behavioral and psychological responses. This view was supported in the current study.

Self-transcendence values were negatively correlated with depression and loneliness. This value dimension emphasizes transcending one's own momentary interests and desires and focuses on those of others. Adolescents who value this dimension, on the one hand, may feel more other-oriented emotions (e.g., empathy, compassion) (Persson and Kajonius, 2016; Leersnyder et al., 2017); such emotions can strengthen the connection between individuals and others (Tamir et al., 2015; Stellar et al., 2017). On the other hand, they may be driven to engage in activities that facilitate building harmony relationships (e.g., helping others) (Davis et al., 2018; Silke et al., 2018); such behaviors can enhance one's purpose in life and improve interpersonal relationships (Nelson et al., 2016) to diminish their negative emotions (Vaillant, 2002). In contrast, self-enhancement was positively correlated with depression. Previous studies have also suggested that these values are rather unhealthy (Bull and Mittelmark, 2008; Bojanowska and Piotrowski, 2018). This value dimension emphasizes the pursuit of self-interest by controlling others and resources or gaining social recognition. Adolescents who endorsed these values may pay more attention to the self and concern about how others think of him or her. As a result, they are vulnerable to ego threats and tend to feel more self-oriented emotions, such as depression, anxiety, and sadness (Leary and Terry, 2012; Twenge, 2015). Such self-oriented emotions are harmful not only to one's mental health but also to meaningful interpersonal relationships. More importantly, Dambrun et al. (2012) hold that adolescents who value this dimension may lead by a “hedonic principle,” that is to say, they were motivated to pursue pleasure and avoid displeasure. However, attaining these pleasures depends on the appearance or disappearance of certain stimuli. If they cannot experience agreeable feelings, it will produce afflictive affects, such as frustration and anger.

In addition, the study found that openness to change values were negatively correlated with depression and loneliness. This dimension emphasizes the independence of thought, action, feelings, and readiness for change (Schwartz, 2015). Adolescents who value this dimension may be motivated by curiosity to search for novel lifestyles and explore new interests, which may be seen as adaptive (Luyckx et al., 2006). This might be because adolescents are expanding their self-identity and focusing on the development of the autonomous self. Based on a functional perspective (Gouveia et al., 2015), endorsing openness to change values will express and satisfy autonomous and independent needs, so adolescents have less depression and loneliness. In contrast, conservation was positively correlated with depression and loneliness. Sortheix and Schwartz (2017) also found that conservation was maladaptive. This dimension emphasizes order, self-restriction, preservation of the past, and resistance to change. Endorsing conservation values may express anxiety and afraid of gaining autonomy (Bojanowska and Piotrowski, 2018). If the autonomy needs cannot be satisfied, adolescents will be depressive and lonely (Emery et al., 2015; Majorano et al., 2015).

### Value Clusters and Mental Health Across Two Regions in China

Four value clusters were identified: *other-focus, self-focus, anxiety-free*, and *undifferentiated*. Each cluster was unique in terms of its dominating values. These value clusters were congruent with previous studies (Ungvary et al., 2017; Bojanowska and Piotrowski, 2018). The *other-focus* values cluster was high in self-transcendence and conservation values, the *self-focus* values cluster was high in self-enhancement and openness to change values, the *anxiety-free* values cluster was high in self-transcendence and openness to change values, and the *undifferentiated* values cluster was a moderate endorsement of the four values dimensions.

Contrary to our hypothesis, an *anxiety-avoidance* values cluster was not identified. An *anxiety-avoidance* values cluster was high in self-enhancement and conservation values. However, according to our descriptive statistical results, the mean scores of self-enhancement and conservation values were −0.42 and −0.16, respectively. This means that the two values were not endorsed much in the current study. In addition, Ungvary et al. (2017) also found that openness to change and self-transcendence values were endorsed more than the other two values during adolescence. Thus, it is difficult to identify an *anxiety-avoidance* value cluster.

There were some differences between the four value clusters in depression and loneliness. First, the current study found that adolescents in the *other-focus* values cluster had higher depression and loneliness scores than adolescents in the *anxiety-free* values cluster. *Other-focus* value clusters primarily promote prosocial behaviors (Schwartz, 2015), and such behaviors make people feel more positive emotions (Vaillant, 2002). However, according to the relations of values to anxiety, conservation values are also accompanied by a self-protection orientation. The relations of values to anxiety also relate to Higgins's (1997) two basic self-regulation systems (Schwartz, 2015). Specifically, the self-transcendence values dimension provides an internalized motivation for prosocial behaviors. In contrast, the conservation values dimension promotes prosocial behaviors to avoid negative outcomes for self. Self-determination theory (Deci and Ryan, 2008) suggests that values that are derived from extrinsic motives are inherently unhealthy. It is possible that desiring such external goals may be forced to engage in high-stress activities (Ryan et al., 1991) and excessive social comparisons (Vansteenkiste et al., 2006). Individuals who endorse the *other-focused* values cluster may be vulnerable to external circumstances in a significant changing environment (Bojanowska and Piotrowski, 2018). If they are unable to cope with it, they may experience negative emotions. However, compared with the *other-focus* values cluster, although the *anxiety-free* values cluster shared the self-transcendence values dimension, it was accompanied by openness to change values dimension, which was related to the autonomy orientation. On the one hand, the dimensions of both self-transcendence and openness to change values that emphasize self-growth are intrinsic motives (Deci and Ryan, 2008; Schwartz, 2015). Such motivations express basic psychological needs of autonomy, relatedness, and competence and have the inherent potential to lead to independent satisfaction. Thus, adolescents in the *anxiety-free* values cluster will benefit both at an individual level (e.g., autonomy and independence) and interpersonal level (e.g., positive relations) (Bojanowska and Piotrowski, 2018). Such benefits are similar to Wayment and Bauer's (2017) quiet ego, which reflects a balance of concerns for the self, and others can promote one's well-being. Hence, adolescents in the *anxiety-free* values cluster feel less depression and loneliness than adolescents in the *other-focus* values cluster.

Second, the study found that adolescents in the *other-focus* values cluster also had higher loneliness scores than adolescents in *the self-focus* values cluster. This result seems slightly odd at first glance because the *other-focus* values cluster emphasizes cooperation, which helps to decrease loneliness, while the *self-focus* values cluster emphasizes self-interest at the cost of relationships. The main task in adolescence is to develop and form self-consciousness. *Self-focus* value clusters primarily regulate how one expresses personal interests and characteristics and meets one's autonomy and independent needs. When these needs are met and self-identity is formed, adolescents will feel less loneliness (Moore and Schultz, 1983; Deng et al., 2015). Thus, the *self-focus* values cluster may be more adaptive than the *other-focus* values cluster during adolescence.

In addition, the study found that adolescents in the *undifferentiated* values cluster had higher loneliness scores than adolescents in the *anxiety-free* values cluster. If one has no clear values, it will lead to some negative feelings, such as emptiness and meaninglessness (Kroger and Marcia, 2011).

Moreover, the study found that adolescents in Shanghai have higher depression than adolescents in Qingdao. This may be because, on the one hand, compared with adolescents in Qingdao, adolescents in Shanghai may be influenced by the fast pace of life and may be forced to accept new things every day. If adolescents cannot deal with the conflicts between novelty stimuli and existing ideas, they may develop mental disorders (e.g., depression, anxiety). On the other hand, a crowded living environment may be a risk factor. People in Hong Kong may feel more depressed because of its compact urban environment and high-rise buildings (Ho et al., 2017). In addition, people in such metropolitan environments were associated with increased amygdala activity when confronted with social stress processing, and the amygdala is related to aversion emotions (e.g., anger, fear, anxiety) (Lederbogen et al., 2011).

Finally, contrary to our hypothesis, there were no significant differences in the values clusters related to depression and loneliness among different regions. On the one hand, although Qingdao is more influenced by Confucian culture—which emphasizes harmonious relationships, advocates humble behavior, and requires adolescents to comply with authority (Chen et al., 2012)—over the past three decades, with economic and social development, China has dramatically morphed into a highly competitive, market-oriented society (Liu et al., 2018). As a result, new social skills such as self-direction, independence, and self-confidence are required for adjustment and success (Liu et al., 2018). Thus, adolescents in Qingdao may endorse *other-focus* values cluster less than before. On the other hand, this result suggests that the relationship between values and mental health is more universal than regionally dependent and suggests that a person-oriented approach is a valid method to understand heterogeneity in mental health (Ungvary et al., 2017).

Overall, the present study combines two approaches to reveal a more accurate portrayal of adolescents' values and mental health and provides some evidence to guide adolescents to establish healthy values. Self-transcendence and openness to change values may be healthier than self-enhancement and conservation values during adolescence. In addition, the *anxiety-free* values cluster may be most adaptive during adolescence, while the *other-focus* values cluster may be less adaptive.

The main findings in the study highlight the key roles of Wong's view that self-transcendence can help improve one's mental health (Wong, 2016). In particular, self-transcendence, on one hand, makes us connect with others. On the other hand, self-transcendence meets our autonomy and curiosity so as to discover the beauty and happiness of life. Further, self-transcendence may include or result in an orientation to the spiritual and the religious, including God (Wong, 2016). To emphasize an important point, COVID-19 is a great challenge for the whole world, it not only threatens human's physical health but also makes people experience unprecedented pressure and anxiety. Wong (2020) also advocated an optimistic view that both the medical and psychological (i.e., self-transcendence) fronts can help us win the prolonged battle with COVID-19. That is to say, in addition to effective medical care, we should also face and accept all pains and pleasures together with others. Furthermore, with the rapid development of modern society and the economy, adults may be so busy pursuing materials gains, power, and self-interests, and the egocentric tendency of adolescents is increasingly obvious (Martin and Sokol, 2011; Shek et al., 2014). Such personal goal and egotism may lead to depression, loneliness, selfishness, and pride and may also damage intimate relationships (Twenge, 2015). That is why Wong (2020) suggests that self-transcendence is needed during COVID-19 and the post-pandemic world to transform suffering into strength and joy. Therefore, if one wants to achieve mature happiness, they should exert potential and practice virtues.

To our knowledge, this is the first study to investigate the contribution of values to adolescents' mental health across two regions in China. The present finding is also the first empirical research to support the importance of existential positive psychology (PP2.0) of self-transcendence among adolescents. The study provides some evidence that helps adolescents establish healthy values to achieve a higher level of mental health.

### Limitations and Future Directions

With the development of society, one's values will change in different periods (Li et al., 2018); thus, one limitation of this study is that the obtained results come from a single cross-sectional study and cannot reveal the developmental trajectories of adolescents' values. Longitudinal studies are required to further verify this hypothesis. Second, although the study found that there were no regional differences between the four value clusters on depression and loneliness, it is necessary to note that our regional groups may differ in socioeconomic status and neighborhoods. Thus, future studies should disentangle the effects of socioeconomic status and neighborhood on the correlation between values and mental health. Third, the study only examines the relationship between values and mental health; however, how values influence mental health is not clear. Future studies can explore the mediating role of different variables, such as self-esteem and emotion regulation.

## Conclusion

In general, the study found that (1) self-enhancement and conservation values correlate positively with mental disorder, self-transcendence and openness to change values correlate negatively with mental disorder; (2) the *anxiety-free* values cluster may be the most adaptive cluster, while the *other-focus* values cluster may be the least adaptive cluster during adolescence; (3) adolescents in Shanghai have higher levels of depression than adolescents in Qingdao. Overall, this study suggests that self-transcendence can help to improve mental health, and also provides some reference for coping with psychological health problems during the COVID-19 pandemic.

## Data Availability Statement

The raw data supporting the conclusions of this article will be made available by the authors, without undue reservation.

## Ethics Statement

The studies involving human participants were reviewed and approved by Ethics Committee of Shanghai Normal University. Written informed consent to participate in this study was provided by the participants' legal guardian/next of kin.

## Author Contributions

PL and XW analyzed the data and wrote the manuscript, they contributed equally to this work. DL designed the study and revised the manuscript. RZ revised the manuscript. HL and JH collected the data. All authors contributed to the article and approved the submitted version.

## Conflict of Interest

The authors declare that the research was conducted in the absence of any commercial or financial relationships that could be construed as a potential conflict of interest.
